# Taurasi DOCG Wines from the Campania Region: A Pilot Study with an AI Approach on a Local Grape Cultivar

**DOI:** 10.3390/metabo15110725

**Published:** 2025-11-06

**Authors:** Daniela Evangelista, Giuseppina Crescente, Giovanni Cascone, Floriana Boscaino, Tanvir Sajed, Mariagrazia Volpe, Vasuk Gautam

**Affiliations:** 1 National Research Council, Institute of Food Sciences, 83100 Avellino, Italy; giuseppina.crescente@cnr.it (G.C.); floriana.boscaino@cnr.it (F.B.); mariagrazia.volpe@cnr.it (M.V.); 2 National Agency for New Technologies, Energy and Sustainable Economic Development, 80055 Portici, Italy; giovanni.cascone@enea.it; 3 Department of Biological Sciences, University of Alberta, Edmonton, AB T6G 2E9, Canada; tsajed@ualberta.ca; 4 Norton Neuroscience Institute, 200 E. Chestnut St., Louisville, KY 40202, USA; vasuk.gautam@nortonhealthcare.org

**Keywords:** Taurasi variety, wine aging, machine learning, K-means, antioxidant capacity, HPLC-UV-DAD, SPME-GC/MS

## Abstract

**Background:** The aging evolution of Taurasi, an *Aglianico*-based DOCG wine from southern Italy, has been comprehensively characterized here for the first time. Known for its high levels of bioactive metabolites with potential health benefits, this variety was analyzed using an integrated analytical and computational approach to explore the evolution of its composition during bottle aging. **Methods:** The analytical framework encompassed physicochemical profiling (density, pH, °Brix) and targeted spectrophotometric analyses for polyphenols (Folin–Ciocâlteu), antioxidant capacity (DPPH, ABTS), and anthocyanins (TAC). The phenolic and volatile fractions were analyzed in detail qualitatively and quantitatively using HPLC-UV-DAD and HS-SPME-GC/MS, respectively. **Results:** The aging process was characterized by a profound phenolic reorganization, with a significant decrease in monomeric phenols and an accumulation of key phenolic acids and stilbenes. The net result of these opposing trends was an overall increase in antioxidant capacity, a significant decrease in total anthocyanins, and an aromatic shift from primary fruity esters to a complex tertiary bouquet. Additionally, machine learning techniques were employed to predict aging-related variations in antioxidant activity and chemical parameters, establishing links between compositional shifts and potential bioactivity. **Conclusions:** This study marks the first-ever investigation of Taurasi *Aglianico* wine aging, offering a novel multidisciplinary approach that provides comprehensive insights into the wine’s chemical evolution. The findings emphasize the potential of Taurasi *Aglianico* in both enological and health-related contexts, highlighting its unique aging dynamics and bioactive potential.

## 1. Introduction

Wine is a traditional alcoholic beverage of significant commercial importance, produced through the total or partial alcoholic fermentation of grape must from *Vitis vinifera* L. plants by yeasts of the *Saccharomyces genus* [1]. Wine is a complex mixture of several hundred compounds, and its chemical composition is highly variable, influenced by numerous environmental factors (such as climate, weather conditions, and soil), by the grape variety, and by the aging process [2]. On average, the main components of wine include water (86%), ethanol (12%), glycerol and polysaccharides or other trace elements (1%), various organic acids (0.4%), polyphenols, and volatile compounds. Although polyphenols constitute only 0.1% of red wine, their impact on its sensory characteristics and aroma composition is substantial [1]. This group encompasses several important families, including flavanols (e.g., catechin), stilbenes (e.g., *trans*-resveratrol), flavonoids (e.g., quercetin), and hydroxybenzoic acids (e.g., gallic acid) [3]. During aging, wine metabolites undergo a series of chemical transformations that play a crucial role in shaping the sensory properties. Anthocyanins, for example, participate in reactions that give rise to more stable oligomeric and polymeric pigments, significantly contributing to variations in color and astringency [4]. As a result, the color of wine gradually shifts, from the bright purple typical of young wines to the lighter brick-red hue characteristic of aged wines [5]. Flavanols are also involved in condensation reactions [4]. In parallel, the modification of volatile compounds during aging is essential for the development of aromatic complexity [6]. These reactions are modulated by both internal (e.g., phenolic content, oxygen, pH, and microbial activity) and external (e.g., temperature, storage materials, humidity, and light exposure) factors [4]. The two primary types of aging are barrel aging and bottle aging, each contributing differently to the evolution of wine metabolites. Barrel aging involves storing wine in wooden barrels, usually oak, where both the wood and controlled oxygen exposure shape the wine’s development. The barrel’s porous structure enables micro-oxygenation, triggering chemical reactions with compounds extracted from the wood. Typically, the maturation period in barrel spans from a few months to over a year, and in some cases, it can extend for several years. During this phase, a two-way exchange occurs between the wine and the barrel, involving volatile and non-volatile compounds that contribute to its aromatic complexity. The type of wood and the time of storage also influence these processes. Furthermore, barrel aging involves other key transformations, such as the condensation of tannins and flavonols, the transfer of aldehydes, and the polymerization of pigments. Moreover, toasting the wood used in barrels leads to the formation of additional aroma compounds, like furans, vanillin, and lactones, although it may also degrade components such as ellagitannins and norisoprenoids. These effects vary with the level of toasting and the oak species used [7,8]. Unlike barrel aging, bottle aging occurs in glass containers sealed with cork stoppers. While cork remains the traditional choice, alternative sealing materials are also used, each with distinct permeability characteristics that affect the wine’s exposure to oxygen. Changes in oxygen exposure affect key reactions, including pigment polymerization, tannin condensation, and the development of off-flavors. Storage in non-ideal conditions, defects in the sealing system, or prolonged aging can promote the accumulation of undesirable substances, which occasionally compromise the wine’s edibility [7]. In addition to these two traditional methods, factors such as container material and environmental conditions (e.g., temperature, humidity, light) also influence wine evolution. The chosen approach is guided by the desired sensory characteristics of the final product. The complexity of these chemical transformations related to aging requires systematic study, particularly in high-quality red wines like Taurasi DOCG. This DOCG, the iconic red wine of Campania, stands out not only for its denomination, but also for its profound aging potential, determined by its unique composition and distinctive terroir. Taurasi production is strictly regulated to safeguard quality and authenticity. According to the official regulations, it must be made with at least 85% *Aglianico* grapes, with the remainder composed of non-aromatic red grape varieties authorized in the province of Avellino. Minimum aging is three years, including at least one year in wooden barrels; for the “Riserva” designation, the requirement is four years, with a minimum of 18 months in barrels. In particular, the DOCG designation reserved for Taurasi requires compliance with several specific conditions. In our case, the production area corresponds to the small village of Sant’Angelo all’Esca in the province of Avellino, where the vineyards are located on hillsides and the climate is typical of the Irpinian hinterland, characterized by wide temperature variations, low rainfall, and limited environmental warming, with average annual temperatures ranging between 10 and 21 °C. Another distinctive feature is the ventilation, which is considerably weaker than that of other, more exposed areas of Campania. It is crucial that, despite its chemical complexity and prestige justifying in-depth analysis, Taurasi remains surprisingly understudied in the literature, particularly concerning its systematic chemical characterization across multiple vintages, compared to other Italian DOCG red wines of similar structure and prestige.

In this pilot study, the first of its kind, we investigate Taurasi wine produced by a world-recognised winery in Campania, focusing on the 2015, 2018, 2021, and 2022 vintages. Our goal is to characterize its compositional and functional parameters and model the effects of bottle aging on chemical parameters, total and specific polyphenolic profile, antioxidant activity, and volatile organic compounds. Recent advances in computational modeling, which have been leveraged in food science to improve product quality [9,10,11], motivate our strategy for Taurasi wine analysis. Therefore, by coupling comprehensive chemical profiling with machine learning-based prediction of bottle-aging dynamics, we introduce an approach not yet applied to Taurasi, thereby addressing a major unmet need in precision enology. The findings will provide new insights into the chemical and sensory features that shape Taurasi’s aging potential, supporting improved winemaking practices, enhanced quality, and the development of wines tailored to specific and sensory profiles, benefiting both producers and consumers.

## 2. Materials and Methods

The red wine samples analyzed in this study belong to the *Aglianico* cultivar, specifically Taurasi, and were kindly provided by Tenuta Cavalier Pepe s.r.l. of Luogosano, Avellino, Campania, Italy. The aging periods investigated include 2015, 2018, 2021, and 2022, here referred to as *T15*, *T18*, *T21*, and *T22*, respectively.

The winemaking process involves the following steps: harvesting, crushing, fermentation, clarification, aging, and bottling. To produce a Taurasi wine, after fermentation, this cultivar requires an initial refinement in steel for 12 months, followed by another 12 months in cement, and finally, aging in Burgundy’s French oak barrels for the last 12 months.

**Figure 1:**
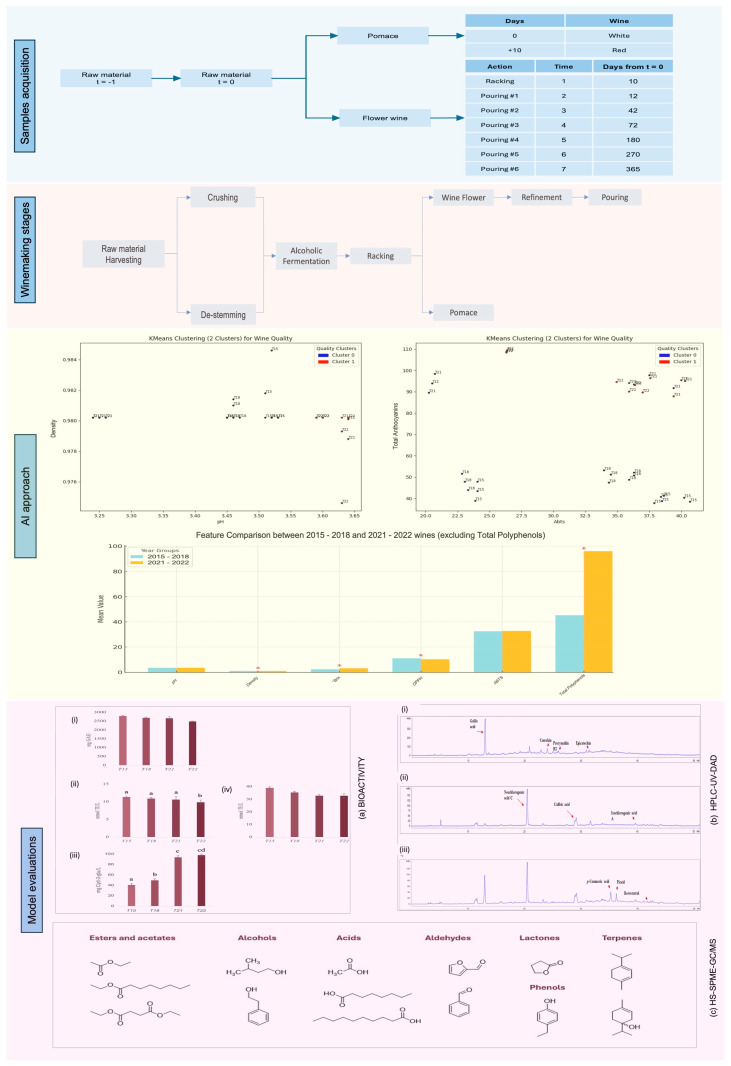
Overview of the study workflow, structured into four main sections: (1) *Sample acquisition*, describing the wine processing monitored throughout the year; (2) *Winemaking stages* corresponding to the collected samples; (3) *AI*-based analytical *approach*; and (4) *Model evaluation* from bioactivity (**a**) and analytical perspectives (**b**,**c**).

The chemical analysis of grapes and wine, especially the study of polyphenols, is a complex process aimed at understanding how these compounds evolve during maturation and aging (Figure 1a). After a year of research, a profile was established detailing the progression of polyphenolic compounds in the analyzed *Aglianico* red wines. This profile included a breakdown of both phenolic acids and anthocyanins, tracking their development from the initial grape stage through the wine’s maturation process (Figure 1b). In this study, wine samples from different vintages were characterized using a multi-analytical approach to investigate temporal changes in composition. High-performance liquid chromatography (HPLC-UV-DAD) was employed to profile phenolic compounds, while volatile compounds were analyzed through headspace solid-phase microextraction coupled with gas chromatography–mass spectrometry (HS-SPME-GC/MS).

### 2.1. Physico-Chemical Parameters

To assess the physico-chemical parameters of the wine samples, several methods were employed. The density was determined using a semi-analytical balance (Crystal 300 CAL, Gibertini; Milan, Italy) and the results were expressed in g/cm^3^. The pH was measured with a digital pH meter (S20 SevenEasy™, Mettler-Toledo; Columbia, MD, USA). Total soluble solids (TSS), expressed in degrees Brix, were quantified using an Abbe refractometer (RM, Exacta + Optech; Munich, Germany).

### 2.2. Determination of Phenols Content

The Total Phenol Content (TPC) was assessed using the Folin–Ciocalteu method, officially recognized by [12] for wines. 0.1 mL of each sample (diluted 10-fold) was combined with 2.3 mL of H_2_O_d_, 0.1 mL of Folin–Ciocalteu reagent (diluted 1:1 with H_2_O_d_, *v*/*v*), and 0.2 mL of Na_2_CO_3_ (20%, *w*/*v*) [13]. The mixture was stirred and left in the dark for 90 min at room temperature. Absorbance was measured at 760 nm using a DU730 UV–Vis spectrophotometer (Beckman Coulter; Milan, Italy). The phenolic content was determined by reference to a gallic acid calibration curve, with results expressed as milligrams of gallic acid equivalents (GAE) per liter.

### 2.3. Antioxidant Activity

The antioxidant activity of wine samples was evaluated based on their ability to neutralize the radical DPPH [2,2-diphenyl-1-picrylhydrazyl] and the radical cation ABTS [2,2′-azinobis-(3-ethylbenzothiazolin-6-sulfonic acid)]. A 0.1 mM DPPH methanol solution was added to the samples (diluted 10-fold) and, after a 20 min incubation, the absorbance was recorded at 517 nm using a spectrophotometer (Synergy HT microplate reader, BioTek, Milan, Italy). ABTS was prepared following the method described by [14]. The solution was then diluted in Phosphate-buffered saline (PBS; pH 7.4) to achieve an absorbance of 0.7 at 734 nm. To each diluted sample (0.01 mL), 0.290 mL of the ABTS solution was added. After a 6 min incubation, the absorbance was measured at 734 nm using a spectrophotometer (Synergy HT microplate reader, BioTek, Milan, Italy). Trolox was used as a positive reference in both antiradical assays. Results were expressed as millimoles of Trolox equivalents (TE) per liter.

### 2.4. Determination of Total Anthocyanin Content

The Total monomeric Anthocyanin Content (TAC) was measured using the differential pH method [15]. Samples were diluted 1:8 with 0.4 M KCl-HCl buffer (pH 1) and 0.4 M sodium acetate buffer (pH 4.5). After incubation in the dark for 15 min, the absorbance of both solutions was measured at 510 nm and 700 nm using a DU 730 UV–Vis spectrophotometer (Beckman Coulter; Milan, Italy). The results were expressed as monomeric anthocyanins as cyanidin-3-glucoside equivalents in mg/L.

### 2.5. Chemical Characterization of Wine Samples

#### 2.5.1. HPLC-UV-DAD Analysis

The HPLC-UV-DAD analysis was carried out using a 1260 Infinity II LC System (Agilent, Santa Clara, CA, USA), equipped with a G7111A quaternary pump and a WR G7115A diode array detector. Separation was achieved with a Poroshell 120 EC-C18 column (150 × 4.6 mm i.d., 4.0 µm particle size, Agilent, Santa Clara, CA, USA) at 30 °C. The mobile phases consisted of water (phase A) and methanol (phase B), both with 0.02% trifluoroacetic acid. Elution was performed using the following gradient: 0–2 min, 0 → 0% B; 2–14 min, 0 → 18% B; 14–24 min, 18 → 25% B; 24–26 min, 25 → 28% B; 26–29 min, 28 → 35% B; 29–45 min, 35 → 58% B; 45–50 min, 58 → 10% B. Phase B was then returned to the initial conditions and equilibrated for 1 min. The total run time was 51 min, with a flow rate of 0.5 mL/min and an injection volume of 10 µL. UV detection was performed at three wavelengths (280, 307, and 320 nm). Retention times and spectral data were compared to standards for compound identification.

#### 2.5.2. Solid Phase Microextraction Gas Chromatography/Mass Spectrometer Analysis of Volatile Compounds (SPME-GC/MS)

Volatile organic compounds (VOCs) were evaluated according to [16], with some modifications. SPME fiber (DVB/CAR/PDMS (divinylbenzene/carboxen/polydimethylsiloxane, thickness 50/30 mm)) was conditioned according to the manufacturer’s recommendations before its first use. To a 20 mL Headspace vial was added 5 mL of wine samples, 3 g of NaCl and octan-3-ol, in hydro-alcoholic solution (1/1, *v*/*v*) at 100 mg L^−1^, as Internal Standard. The solution was homogenised with a vortex shaker and then loaded onto a Gerstel autosampling device. The program consisted of swirling the vial at 250 rpm for 5 min at 40 °C, inserting the fiber into the headspace for 30 min at 40 °C as the solution was swirled again and transferring the fiber to the injector for desorption at 240 °C for 30 min. Gas chromatography analysis was performed using a 7890 Agilent GC system coupled to an Agilent 5975 inert quadrupole Mass Spectrometer (MS) equipped with a Gerstel MPS2 autosampler. The capillary column used was an HP-Innowax (Supelco®, Bellefonte, PA, USA) (30 m × 0.25 mm i.d. × 0.50 µm film thickness), and the carrier gas was Helium. Splitless injections were used. The initial oven temperature was set at 40 °C for 1 min. The temperature was increased in four steps, 40–60 °C at 2 °C min^−1^; 60–150 °C at 3 °C min^−1^, 150–200 °C at 10 °C min^−1^ and 200–240 °C at 25 °C min^−1^, and the final temperature was maintained for 7 min. The injector, the quadrupole, the source and the transfer line temperature were maintained at 240 °C, 150 °C, 230 °C and 200 °C, respectively. Electron ionization mass spectra in full-scan mode were recorded at 70 eV electron energy in the range 40–300 amu. Identification of VOCs was achieved by comparing mass spectra with the Nist library (NIST 20). The data are expressed as relative peak area respect to internal standard (RAP). Blank experiments were carried out in two different modalities: blank fibers and blank empty vials. All the analyses were performed in duplicate for each biological replicate, and the results were expressed as mean value ± standard deviation [16].

### 2.6. ML Computational Tools

All analyses performed on this cultivar, from a computational point of view, were developed using an Apple M2 Ultra, 128 GB RAM on Weka platform v.v. 3.8.6, compared with Jupyter notebook with Python kernel based on Python, v.v. 3.12.3.

### 2.7. Data Pre-Processing and Exploratory Data Analysis

The Taurasi wine has six properties which explains the overall composition of wine and these properties combined provide an overall picture of the quality of wine. One of the main data points or the properties is the physiochemical properties that is named as “total anthocyanins”. It was also confirmed by multiplicity control using Benjamini–Hochberg FDR (BH-FDR) [17], and using this methodology the group differences were also found strongest for Anthocyanin total, °Brix, and DPPH (q < 0.05), whereas density and Polifenoli totali were nominally significant (*p* < 0.05) but did not survive FDR; pH and ABTS showed no differences (Table S1). These results were consistent under Welch/nonparametric sensitivity tests. Since there are six properties that define the quality of the wine and prepare the data for machine learning analysis. We have converted the output to a binary classification problem where each wine is either “good quality” or not. The comparison plot for two classes of wine quality is shown in Figure 1.

For many ML models, a resampling technique like K-fold Cross-Validation Synthetic Minority Oversampling Technique (SMOTE) [18] may be required if the data was extremely imbalanced, however in this case, the data seems to be balanced. First, the duplicate values present in the dataset are dropped to perform data cleaning. After that, the index is reset to make the data uniform. We normalized the data by changing it so that its distribution will have a mean of 0 and a standard deviation of 1 to prepare it for modeling. Standardizing the data is crucial to equating the data’s range and preventing bias. The ML models were trained using this dataset.

### 2.8. Features Selection and Machine Learning Analysis

We first reduced data dimensionality to summarize the multivariate structure while retaining most of the variance. To that end, features were standardized and subjected to principal component analysis (PCA). The decomposition identified orthogonal directions of maximal variability; the first two principal components explained the majority of total variance and were retained for downstream analysis. Samples were then projected into this two-dimensional space, providing an interpretable representation for visual inspection and unsupervised modeling. To ensure the embedding was not driven by a few extreme variables, we confirmed that loadings were broadly distributed across chemical classes and that the principal directions were stable under leave-one-sample sensitivity analyses. To probe latent group structure, K-means clustering [19] was applied to the PCA scores and, in a complementary analysis, to the full standardized feature matrix. The number of clusters was selected using the elbow criterion on the within-cluster sum of squares, balancing parsimony with separation; results were robust to multiple random initializations. We examined whether clusters aligned with anticipated sample attributes—most notably vintage (pre-2021 vs. post-2021)—and whether structure emerged that was more consistent with quality-related differences irrespective of year. Concordant patterns across the PCA space and the full feature space suggested that clustering was not an artifact of the dimensionality reduction. Cluster discriminants were evaluated statistically. Mean values of prespecified analytes—density, °Brix, DPPH, and Anthocyanins—were compared across clusters using independent t tests or one-way ANOVA, as appropriate, with *p* < 0.05 denoting statistical significance. Each of these features differed significantly between clusters, indicating that they contributed materially to the observed partitioning; effect directions were consistent with a priori expectations for markers of ripeness (°Brix), extract/structure (Anthocyanins), and redox status (DPPH). Operationalizing wine quality a priori, we considered higher °Brix and anthocyanins, an appropriate (not excessive) density, and a favorable antioxidant profile captured by DPPH as indicative of better quality; clusters enriched for this profile were interpreted as representing comparatively higher-quality wines. For presentation, effect magnitudes and directions were summarized in bar plots with significance annotations to highlight markers most associated with cluster separation, alongside PCA score plots colored by cluster to visualize between-group dispersion.

### 2.9. Statistical Analysis

For each of the four vintages, three independent bottles were analyzed. Measurements were performed in triplicate for each bottle, and the resulting data are presented as mean ± standard deviation (SD). Statistical analysis was performed using SigmaPlot 15.0 (SPSS Inc., Norman Nie Dale Bent, Hadlai “Tex” Hull, Chicago, IL, USA) software. Differences between mean values were assessed using the Tukey test (95% confidence level; *p* < 0.05).

**Figure 2:**
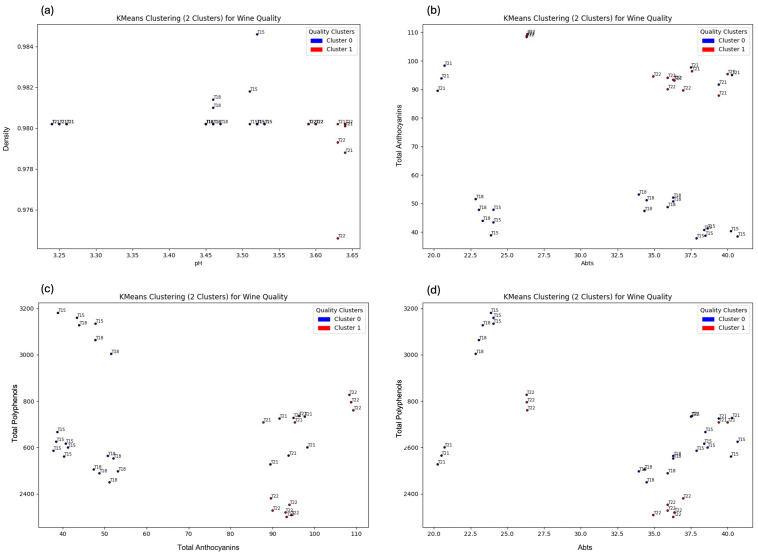
K-means clustering for wine quality: (**a**) density vs. pH; (**b**) total anthocyanin vs. ABTS; (**c**) total phenols vs. total anthocyanin; (**d**) total phenols vs. ABTS.

From a ML side, after performing clustering on the wine samples, the dataset was segmented into distinct groups, allowing for a deeper analysis of their relationships with factors such as vintage year and wine quality (Figure 2). The primary goal was to determine whether key features could differentiate between these groups. To assess this, statistical tests, including independent t-tests and one-way ANOVA, were applied to evaluate the significance of features such as density, °Brix, DPPH, and Anthocyanins between the different clusters. A significance threshold of 0.05 was used to determine statistical significance. When the *p*-value was less than 0.05, the null hypothesis was rejected, indicating that these features significantly contributed to distinguishing the clusters. The results from these statistical tests revealed that certain features, particularly density, °Brix, DPPH, and Anthocyanins, exhibited considerable differences between the clusters. This finding suggests that these properties are vital in differentiating high-quality wines from lower-quality ones, and their roles are essential for wine classification. To further validate the model’s performance and ensure its robustness, K-fold cross-validation [20] was employed. This technique, which divides the data into multiple subsets for training and testing, was especially important for evaluating the generalizability of the model. Given the balanced nature of the dataset, K-fold cross-validation [20] provided a reliable assessment of the model’s ability to predict wine quality without overfitting, ensuring that the results would be applicable to new, unseen data.

## 3. Results

### 3.1. Impact of Aging on the Physicochemical Profile of Aglianico Wines

The physicochemical parameters of *Aglianico* wine samples from four vintages (2015, 2018, 2021, and 2022) provided a clear overview of the wine’s evolution during aging. A summary of density, pH, and °Brix values across the vintages is presented in Table 1.

The youngest sample, *T22*, exhibited the lowest density (0.975 ± 0.004 g/cm^3^), the highest pH (3.608 ± 0.020), and the highest °Brix value (3.228 ± 0.412). Conversely, the older sample, *T15*, showed a slightly higher density (0.982 ± 0.002 g/cm^3^), a lower pH (3.520 ± 0.007), and a lower sugar content (2.250 ± 0.395). Density varied minimally between vintages, with no statistically significant effect of aging and no consistent age-related trend, consistent with what has been reported for other red wines (e.g., Merlot) [21]. Although the variations were marginal and not significant, a slight age-related increase would be consistent with the gradual formation of polymeric pigments and tannin-protein complexes [4]. Conversely, pH differed between vintages (different superscript letters in Table 1), describing a non-linear trend over time: pH decreased from 3.520 ± 0.007 in *T15* to 3.379 ± 0.193 in *T21*, followed by a sharp increase in *T22* (3.608 ± 0.020). Red wines generally exhibit pH values between about 3.3 and 3.7 [22]. Increases in *T22* may be related to multiple factors, including vintage-specific climatic conditions and changes in acid composition during winemaking and storage. Climatic conditions can influence grape composition and, consequently, the chemistry of wine, with increases in pH and decreases in acidity levels [23]. In our analysis, climate data indicated that, although average temperatures during grape ripening were similar across the years considered (T: 19.20 °C in 2022 vs. 19.28 °C in 2021), average precipitation was markedly higher in 2022, especially in August-September (3.79 and 5.20 vs. 0.56 and 0.81 mm day^−1^; October 1.70 vs. 1.51 mm day^−1^) [24]. These differences suggested that water availability during the ripening–harvest period may have exerted a stronger influence on pH than temperature in this dataset. In addition, modifications in acid composition, such as precipitation of tartaric acid and metabolism of malic acid by yeasts and lactic acid bacteria, could also contribute to the observed trend [22,25]. Sugar content, measured in °Brix, showed a decreasing trend with aging (different superscript letters in Table 1). During the aging of red wine, residual sugars can participate in small chemical transformations that influence the sensory characteristics of the wine. Initially, during alcoholic fermentation, yeasts metabolize grape-derived sugars into ethanol and carbon dioxide; after fermentation, a small fraction of residual sugars persists [26]. These residual sugars can engage in chemical reactions, such as the Maillard reaction, in which sugars interact with amino acids to form complex flavor compounds [27]. Although the Maillard reaction happens more quickly at higher temperatures, it can still take place, although at a slower rate, at cellar temperatures [28]. The decline from 3.228 ± 0.412 in *T22* to 2.250 ± 0.395 in *T15* is consistent with more complete fermentation of sugars over time, as well as the possible formation of more complex compounds through Maillard reactions [29].

Overall, vintage-related differences were determined by pH and °Brix, while density remained essentially unchanged.

### 3.2. Bioactivity of Wine Samples

**Figure 3:**
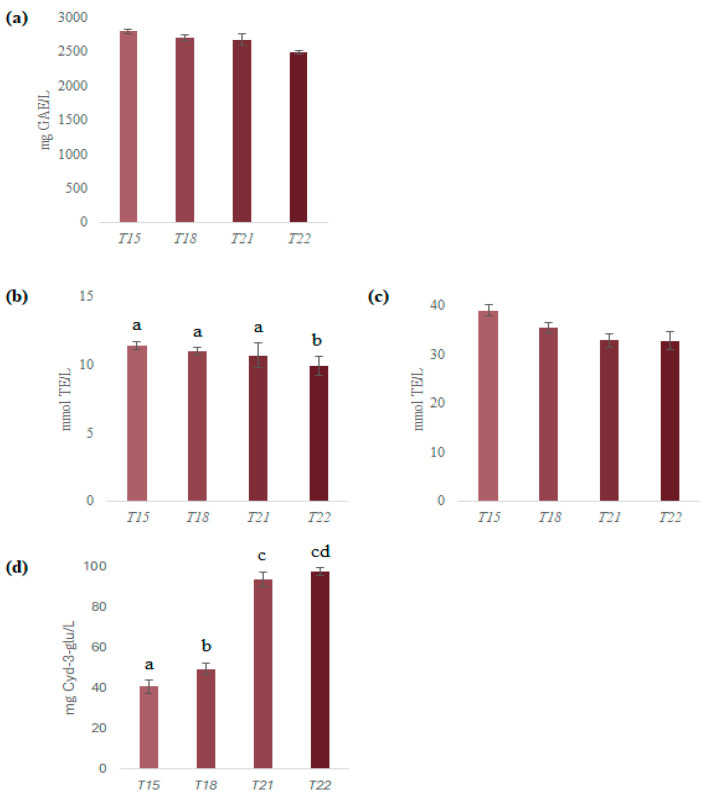
Total Phenol Content (TPC, expressed as mg of gallic acid equivalent [GAE] per L of sample; panel (**a**)); antiradical activity (expressed as mmol of Trolox equivalent [TE] per L of sample) towards DPPH radical (panel (**b**)) and ABTS radical cation (panel (**c**)); Total Anthocyanin Content (TAC, expressed as mg of cyanidin-3-glucoside equivalent per L of sample; panel (**d**)). Different letters indicate significant differences (*p* < 0.05). The absence of letters indicates non-significant differences between samples.

The chemical composition and bioactive properties of red wines are significantly influenced by various factors, among which aging plays a fundamental role. Phenolic compounds, responsible for key sensory attributes and antioxidant potential, undergo dynamic changes over time due to complex chemical reactions such as oxidation, polymerization and precipitation [4]. The evaluation of TPC, antioxidant activities (DPPH and ABTS assays) and TAC in *Aglianico* wine samples from different vintages highlighted variations associated with wine aging. The acquired data are shown in graphed in Figure 3. Specifically, TPC did not show statistically significant differences between ages (*p* = 0.061), with the lowest TPC recorded in *T22* (2485.51 ± 30.16 mg GAE/L) and the highest in *T15* (2791.88 ± 36.16 mg GAE/L). Although the differences between vintages did not reach statistical significance, the slightly higher TPC in older samples is consistent with the stabilization of polyphenols over time, likely involving the polymerization and precipitation of larger tannins, as has been reported for red wine aging [30,31].

Similar trends were observed for antioxidant activities measured by DPPH and ABTS assays. For DPPH, a significant aging effect was detected (one-way ANOVA *p* < 0.001), with values increasing from 9.87 ± 0.68 (*T22*) to 11.34 ± 0.29 (*T15*). Tukey’s post hoc analysis indicated *T22* < *T15*, *T18*, and *T21* (*p ≤* 0.047), while the latter three showed no significant differences. Although ABTS tended to increase between vintages, the differences were not significant (one-way ANOVA *p* > 0.05); values ranged from 32.83 ± 1.90 to 39.06 ± 1.12 mmol TE/L. The observed increase in antioxidant activity in aged wines can be attributed to several factors. Aging leads to changes in polyphenolic composition, including a reduction in free anthocyanins and an increase in highly polymerized proanthocyanidins [32]. These polymeric compounds, due to their extended structures, can stabilize free radicals through electron delocalization, thereby enhancing the wine’s antioxidant capacity. Additionally, there is a strong correlation between TPC and antioxidant activity in wines. Conversely, TAC displayed a notable increase in all vintages (one-way ANOVA *p* < 0.001), peaking in younger wines such as *T22* (97.90 ± 2.10 mg/L) compared to *T15* (40.83 ± 3.14 mg/L). Tukey’s post hoc analysis confirmed *T22* ≈ *T12* > *T18* > *T15* (*p* ≤ 0.004 for all pairs except *T22* vs. *T21*, *p* = 0.356).

In these young, anthocyanin-rich wines, self-association and copigmentation are key mechanisms. These interactions shift the apparent stability of the red flavylium cation toward slightly higher pH values and increase the proportion of quinoid bases. This structural stabilization supports color expression and enhances apparent anthocyanin retention at typical wine pH (approximately 3–4), counteracting the general pH-induced decline in monomer stability [33,34]. Therefore, the higher TAC observed at *T22* was mainly explained by the combined effect of wine age/concentration and copigmentation, rather than by an intrinsic increase in monomer solubility at higher pH.

### 3.3. Chemical Characterization

#### 3.3.1. Polyphenol Evolution in Wine Samples Through Vintages by HPLC-UV-DAD

**Figure 4:**
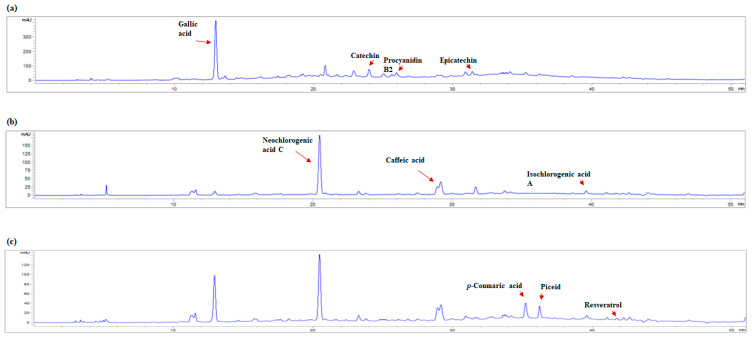
Representative HPLC-UV chromatograms of a Taurasi wine sample at 280 (panel (**a**)), 320 (panel (**b**)), and 307 nm (panel (**c**)). The main peaks are noted.

The polyphenolic composition of Taurasi wines from *Aglianico* grapes underwent marked transformations during aging, as observed in the four vintages analyzed by HPLC-UV/DAD. From the youngest wine (*T22*) to the oldest (*T15*), a clear evolution of the key polyphenolic compounds was evident (Figure 4 and Table S2). 

The youngest vintage (*T22*) showed significantly elevated levels of monomeric flavan-3-ols, with catechin (96.96 ± 0.27 mg/L) and epicatechin (58.74 ± 4.46 mg/L), along with significant concentrations of procyanidin B2 (56.22 ± 0.31 mg/L). As the wine aged, these compounds gradually decreased, with catechin falling to 46.68 ± 2.45 mg/L, epicatechin to 30.95 ± 0.47 mg/L, and procyanidin B2 to 39.89 ± 0.67 mg/L in the oldest sample (*T15*). This steady decrease is consistent with the known polymerization and precipitation mechanisms that occur during aging, leading to the formation of larger polymeric tannins and the loss of monomeric units [35,36]. A similar trend was observed for neochlorogenic acid C, which showed high stability in younger wines (97.46 ± 0.10 mg/L in *T22* and 96.44 ± 0.50 mg/L in *T21*), before a sharp decline in older vintages (51.67 ± 0.21 mg/L in *T18* and 59.05 ± 0.60 mg/L in *T15*). Hydroxycinnamic acids, especially caffeic acid and *p*-coumaric acid, exhibited slightly different behavior. While their absolute concentrations were lower in the younger vintage (2.81 ± 0.56 mg/L and 0.81 ± 0.25 mg/L, respectively), they increased in the older samples, reaching 8.83 ± 0.02 mg/L (caffeic acid) and 2.73 ± 0.41 mg/L (*p*-coumaric acid) in *T15*. This could suggest a progressive release from bound forms or hydrolysis reactions occurring during long-term maturation [37]. Gallic acid showed a gradual increase from 67.25 ± 0.03 mg/L in the youngest vintage (*T22*) to 94.49 ± 1.08 mg/L in *T15*. This increase is consistent with oxidative degradation and precipitation processes associated with hydrolysable tannins during bottle aging [38]. Interestingly, isochlorogenic acid A, absent in *T22*, appeared in detectable amounts in older wines, reaching up to 5.30 ± 0.04 mg/L in *T15*. For stilbenes, resveratrol was lowest in *T22* (0.17 ± 0.01 mg/L) and slightly higher, but without significant differences, in older vintages (*T21*: 0.26 ± 0.02 mg/L; *T18*: 0.29 ± 0.00 mg/L; *T15*: 0.29 ± 0.06 mg/L). Conversely, piceid was significantly higher only in the youngest wine (*T22*: 5.85 ± 0.10 mg/L) and comparable in older vintages (*T21*: 3.55 ± 0.13 mg/L; *T18*: 3.21 ± 0.29 mg/L; *T15*: 3.59 ± 0.08 mg/L). This opposite trend is consistent with the hydrolysis of piceid (resveratrol-glucoside) to resveratrol during wine aging, via the *β*-glucosidase activities of microorganisms participating in malolactic fermentation [39,40]. Finally, vanillic acid showed a modest but steady decrease, from 2.29 ± 0.01 mg/L in the younger wine to 1.55 ± 0.16 mg/L in the older one. While barrel aging facilitates the diffusion extraction of oak-derived phenols, including vanillic acid, it also allows a slow and continuous influx of oxygen through the wood and structural cracks. This influx of oxygen triggers oxidative transformations within the wine matrix. In our specific aging regime (oak contact limited to the last 12 months), this suggests that an initial extraction phase during barrel contact is subsequently replaced, over time, by oxygen-mediated reactions that reduce the freely detectable vanillic acid pool. Therefore, the observed decline is consistent with the combined effects of migration from wood into wine and oxidative consumption under micro-oxygenated conditions in the barrel, rather than extraction alone [41].

#### 3.3.2. HS-SPME-GC/MS Analysis of Wine Samples

The volatile profile of wines is influenced by multiple factors, including the grape variety, geographical origin, climatic conditions, vintage, viticultural practices, wine-making techniques, and aging or storage conditions. The SPME-GC/MS analysis enabled the identification of fifty volatile compounds belonging to different chemical classes: esters and acetates (21), alcohols (10), acids (7), sulfur compounds (2), aldehydes (3), ketones and lactones (2), phenols (2), and terpenes (3) (Table S3). Among these, esters and acetates, along with alcohols, represented the most abundant classes. Ethyl esters are synthesized primarily during yeast fermentation through enzymatic reactions involving grape-derived precursors and the ethanolysis of acyl-CoA, which is formed during the synthesis or degradation of fatty acids. Their concentrations are strongly influenced by factors such as the yeast strain, fermentation temperature, aeration, and sugar content. The predominant ethyl esters identified were ethyl hexanoate, ethyl octanoate, and ethyl decanoate. Acetates are formed through the reaction of acetyl-CoA with higher alcohols derived from the degradation of amino acids or carbohydrates [42]. The main acetates detected were ethyl acetate, diethyl succinate, and isoamyl acetate. Higher alcohols are produced during yeast fermentation through amino acid metabolism or the reduction of related aldehydes [16,42,43]. Isoamyl alcohol was one of the main compounds detected. Acidic compounds, produced enzymatically during fermentation, constitute an important group of factors that contribute to aroma formation. The most abundant acids are acetic acid and octanoic acid. Sulfur compounds, such as methionol, derive from the amino acid methionine [42]. Finally, terpenes, varietal aromatic compounds that depend mainly on the grape variety, such as *α*-terpinene, terpinen-4-ol and *α*-terpineol, were detected. The results are consistent with previous literature, confirming that esters generally decrease with aging. Within the ester family, ethyl esters (e.g., ethyl hexanoate, ethyl octanoate, ethyl decanoate) decreased over time, while lactate-derived esters (ethyl lactate, amyl lactate) and diethyl succinate increased (Table S3). For example, ethyl hexanoate decreased by approximately 62% from (*T15*) to (*T22*) (914.03 → 346.94 RAP; Table S3), while diethyl succinate increased by approximately 10.6-fold over in the same period (296.03 → 3126.83 RAP; Table S3). Ethyl acetate concentration also tends to increase over time; although small amounts are produced by yeast during fermentation, larger amounts are formed through the oxidative activity of acetic acid bacteria, particularly during barrel aging. Higher alcohols (e.g., isoamyl alcohol) typically show minimal changes during aging. Consistently, isoamyl alcohol varied by approximately 10.6% from (*15*) to (*T22*) (3161.05 → 2827.52 RAP; Table S3). Acidic compounds tend to show an increase during maturation. For example, acetic acid approximately doubled (166.73 → 334.82 RAP) and octanoic acid increased approximately 3.65-fold (45.61 → 166.27 RAP) from (*T15*) to (*T22*) (Table S3). Sulfur compounds (e.g., methionol) typically decrease with aging. In our data, methionol decreased from 30.43 to 11.79 RAP between (*T15*) and (*T22*) (Table S3). During maturation, additional volatile compounds such as furfural, benzaldehyde, 5-hydroxymethylfurfural, 2-methoxy-4-ethylphenol, and 4-ethylphenol are formed, likely as a result of interactions with oak wood during barrel aging. For example, furfural was not detected in (*T15*) and reached 14.79 RAP in (*T22*) (Table S3). Finally, terpenes decreased during the aging process, consistent with previous studies [6,44]. Total terpenes decreased from 22.99 RAP in (*T15*) to being undetectable in (*T22*) (Table S3). Both esters and acetates play a key role in the overall aroma of wine, contributing to pleasant fruity and floral notes. In line with these chemical trends, the attenuation of ethyl esters is consistent with a reduction in fruity attributes associated with ethyl hexanoate (pineapple), ethyl octanoate (apricot), ethyl decanoate (apple), and isoamyl acetate (banana). Conversely, the increase in diethyl succinate aligns with floral and tropical fruit nuances. The increase in acetic acid (pungent, vinegar) and octanoic acid (fatty, cheesy) supports the corresponding sensory notes, while wood-derived compounds (furfural (almond, baked bread), benzaldehyde (almond), 5-hydroxymethylfurfural (caramel), 2-methoxy-4-ethylphenol (spicy, smoky bacon), and 4-ethylphenol (smoky)) explain the observed almond, baked bread, caramel, spicy/smoky bacon, and smoky characteristics. The decline in terpenes corresponds to the attenuation of attributes related to *α*-terpinene (citrus, woody), terpinen-4-ol (nutmeg, woody) and *α*-terpineol (floral, lilac type).

## 4. Discussion

This pilot study reports the first investigation of Taurasi wine aging using an integrative framework combining chemical profiling with computational modeling. Subsequent studies will enlarge the sample set, include sensory evaluation for independent validation, and systematically evaluate machine-learning models in adequately powered cohorts. This current work seeks to establish an initial, integrative profile for Taurasi wine, which has not been systematically characterized in the literature. Our objective is to provide data that can bridge basic compositional insights with potential applications, including the generation of hypotheses relevant to dietary guidance and preventive health.

A detailed analysis of wine aging, combining physicochemical, spectrophotometric, and chromatographic data, reveals the intricate and interconnected chemical transformations that define the aging trajectory of this wine. The aging state of wine can be decoded by a composite signature of its fundamental physicochemical properties. Increased density traces the evolution of the macromolecular structure through phenolic polymerization, while decreasing °Brix levels may be consistent with the development of aromatic complexity through pathways such as the Maillard reaction. The nonlinear pH trend highlights the crucial role of the vintage’s initial conditions, highlighting that each harvest possesses a unique chemical signature that guides its long-term evolution. The transformation of phenolic compounds, quantitatively tracked by HPLC-UV-DAD, is central to this aging process. A clear trend emerged: compounds abundant in young wines, particularly flavan-3-ol monomers (catechin, epicatechin), procyanidin B2, and neochlorogenic acid, steadily decreased over time. Conversely, the aging process promoted an enrichment of other key phenolic compounds. The concentration of phenolic acids, including gallic, caffeic, and *p*-coumaric acids, as well as the stilbene resveratrol, increased in older vintages, an enrichment that not only contributes to the complexity and final stability of the wine but also enhances its overall nutraceutical profile. In parallel, the structural evolution is consistent with the observed increase in TPC and higher antioxidant activity in older vintages, as polymeric structures may exhibit greater free radical scavenging capabilities. The significant decline in TAC further exemplifies this trend toward monomer reduction, a key process that drives the wine’s chromatic evolution from youthful ruby tones to the characteristic brick and garnet hues of maturity. Simultaneously, the wine’s aromatic profile undergoes a profound metamorphosis. Initial fruity and floral notes, driven primarily by esters formed during fermentation, gradually diminish over time due to hydrolysis. This decline gives way to a more complex tertiary bouquet, with the evolution of sulphur compounds, acids, and oak-derived molecules that further contribute to this sophisticated aromatic signature and its potential use in healthy diet. However, any consideration of incorporating Taurasi wine within a health-conscious diet is exploratory and intended to complement, not replace, existing medical and nutritional practices: definitive conclusions will require larger prospective studies.

## 5. Conclusions

The novelty of this research lies in applying ML-driven models to the aging process of Taurasi wine with a unique approach not previously explored in winemaking studies. However, this novelty also represents a limitation: there are no directly comparable studies, particularly those integrating density, °Brix, DPPH, and anthocyanins, to which our results can be compared. Since this is one of the first studies, and in the absence of standardized reference datasets and harmonized protocols for Taurasi, cross-study comparisons remain to be further explored and examined. Accordingly, our ML-based quality-related inferences should be corroborated by sensory evaluations and larger cohort datasets.

To build upon this foundational work and address these challenges, future efforts will focus on refining our analytical approach. The development of statistical classification tools will enable more precise predictions of wine aging outcomes based on initial conditions, and these analytical advances will help validate our current hypotheses while revealing new insights into the relationships between phenolic composition, volatile profiles, and aging dynamics. This integrated framework outlines a path to precision winemaking, supporting the management of wine style and quality over time.

## Figures and Tables

**Table 1:** Density, pH, and °Brix values of *Aglianico* wine samples across different vintages.

Sample	Density	pH	°Brix
*T15*	0.982 ± 0.002	3.520 ± 0.007^ab^	2.250 ± 0.395^a^
*T18*	0.979 ± 0.004	3.457 ± 0.007^bc^	2.428 ± 0.593^ab^
*T21*	0.979 ± 0.002	3.379 ± 0.193^c^	3.072 ± 0.926^bc^
*T22*	0.975 ± 0.004	3.608 ± 0.020^a^	3.228 ± 0.412^c^

Data are expressed as mean ± SD. Within each column, means with different superscript letters indicate significant differences (*p* < 0.05). The absence of letters indicates non-significant differences between samples.

## Data Availability

The original contributions presented in this study are included in the article/Supplementary Materials. Further inquiries can be directed to the corresponding author.

## Supplementary Materials

The following supporting information can be downloaded at: https://www.mdpi.com/article/10.3390/metabo15110725/s1, Table S1: FDR value for statistical significance of features; Table S2: HPLC-UV-DAD data of the compounds identified in the wine samples; Table S3: Volatile organic compounds (VOCs) identified by solid phase microextraction/gas chromatography-mass spectrometry in *Aglianico* wine.
