# Supplementary material for: Taurasi DOCG Wines from the Campania Region: A Pilot Study with an AI Approach on a Local Grape Cultivar
Source: Metabolites. 2025 Nov 6;15(11):725. doi: 10.3390/metabo15110725 (PMC12654730; doi:10.3390/metabo15110725)
Supplement: Supplementary file 1 [file metabolites-15-00725-s001.zip › metabolites-3906248-supplementary.pdf]

Supplementary Materials

Table S1. FDR value for statistical significance of features.

Feature	t_statistic	p_value	q_value_FDR	significant_FDR_0.05
pH	0.1171671	0.907417044	0.92057623	FALSE
Density	-2.176707806	0.036541135	0.062563876	FALSE
Brix	4.007904774	0.000316565	0.000854807	TRUE
DPPH	-3.956893765	0.000366346	0.000854807	TRUE
Abts	0.100451073	0.92057623	0.92057623	FALSE
Total Anythocyanin	25.34083645	1.17E-23	8.19E-23	TRUE
Total Polyphenols	-2.084596758	0.044688483	0.062563876	FALSE

Table S2. HPLC-UV-DAD data of the compounds identified in the wine samples.

Rt (min)	Compound	max	*T15* [mg/L]	*T18* [mg/L]	*T21* [mg/L]	*T22* [mg/L]
12.935	Gallic acid	280	94.49 ± 1.08^{a}	89.64 ± 2.20^{b}	80.04 ± 0.02^{c}	67.25 ± 0.03^{d}
20.455	Neochlorogenic acid C	320	59.05 ± 0.60^{a}	51.67 ± 0.21^{b}	96.44 ± 0.50^{c}	97.46 ± 0.10^{c}
23.963	Catechin	280	46.68 ± 2.45^{a}	57.70 ± 1.31^{b}	87.13 ± 1.22^{c}	96.96 ± 0.27^{d}
25.923	Procyanidin B2	280	39.89 ± 0.67^{a}	45.90 ± 0.06^{b}	46.58 ± 0.28^{b}	56.22 ± 0.31^{c}
27.923	Vanillic acid	280	1.55 ± 0.16^{a}	1.65 ± 0.19^{a}	2.24 ± 0.08^{b}	2.29 ± 0.01^{b}
28.830	Caffeic acid	320	8.83 ± 0.02^{a}	8.52 ± 0.04^{a}	2.37 ± 0.04^{b}	2.81 ± 0.56^{b}
31.379	Epicatechin	280	30.95 ± 0.47^{a}	31.49 ± 0.13^{a}	41.65 ± 1.99^{b}	58.74 ± 4.46^{c}
35.208	*p*-Coumaric acid	307	2.73 ± 0.41^{a}	1.93 ± 0.33^{a}	1.08 ± 0.14^{b}	0.81 ± 0.02^{b}
36.210	Piceid	307	3.59 ± 0.08^{a}	3.21 ± 0.29^{a}	3.55 ± 0.13^{a}	5.85 ± 0.10^{b}
39.589	Isochlorogenic acid A	320	5.30 ± 0.04^{a}	4.68 ± 0.03^{b}	0.93 ± 0.05c	nd
41.740	Resveratrol	307	0.29 ± 0.06^{a}	0.29 ± 0.00^{a}	0.26 ± 0.02^{a}	0.17 0.001^{b}

Results are expressed as milligrams per liter (mg/L ± SD). Different superscript letters within a row indicate statistically significant differences between samples ($p < 0.05$). Abbreviations: rt, retention time; nd, not detected.

Table S3. Volatile organic compounds (VOCs) identified by solid phase microextraction/gas chromatography-mass spectrometry in *Aglianico* wine.

	T15	*T18*	*T21*	*T22*
**Esters and acetates**				
Ethyl acetate	1265.93 ± 255.37^{a}	1121.93 ± 167.77^{a}	2706.76 ± 543.45^{b}	2641.04 ± 367.25^{b}
Ethyl propanoate	11.36 ± 1.62	10.86 ± 1.90	10.29 ± 1.81	9.17 ± 1.78
Ethyl isobutanoate	238.64 ± 16.03^{a}	186.91 ± 29.82^{b}	58.84 ± 4.37^{c}	31.04 ± 2.13^{d}
Ethyl butanoate	49.25 ± 3.12^{a}	56.30 ± 10.17^{ab}	68.80 ± 10.99^{bc}	79.35 ± 4.99^{c}
Ethyl 2-methylbutanoate	12.22 ± 1.63^{a}	27.11 ± 4.63^{b}	64.01 ± 13.87^{c}	81.41 ± 8.63^{d}
Ethyl isovalerate	21.52 ± 1.73^{a}	52.96 ± 10.08^{b}	100.47 ± 21.61^{c}	109.46 ± 13.76^{d}
Isoamyl acetate	460.23 ± 101.95^{a}	468.73 ± 24.91^{a}	353.57 ± 32.53^{b}	210.44 ± 16.76^{c}
Ethyl hexanoate	914.03 ± 48.80^{a}	822.61 ± 47.52^{b}	449.65 ± 17.47^{c}	346.94 ± 50.76^{d}
Ethyl heptanoate	28.83 ± 1.84^{a}	13.59 ± 2.00^{b}	28.51 ± 2.62^{a}	20.26 ± 2.52^{c}
Ethyl lactate	134.53 ± 27.81^{a}	101.21 ± 10.31^{a}	395.18 ± 45.25^{b}	553.33 ± 75.06^{c}
Ethyl 2-hydroxy-3-methylbutanoate	78.51 ± 6.60^{a}	nd^{b}	nd^{b}	nd^{b}

Ethyl octanoate	3636.12 ± 184.55[a]	2567.08 ± 331.38[b]	1523.09 ± 275.79[c]	1104.90 ± 37.85[d]
Ethyl nonanoate	15.67 ± 1.22[a]	14.28 ± 0.35[ab]	13.36 ± 2.00[b]	3.25 ± 0.20[c]
Amyl lactate	nd[a]	19.91 ± 3.10[b]	54.35 ± 9.92[c]	70.43 ± 8.19[d]
Ethyl decanoate	921.83 ± 117.17[a]	438.35 ± 65.29[b]	358.23 ± 27.11[b]	338.38 ± 33.01[b]
Isoamyl octanoate	17.64 ± 2.91[a]	12.93 ± 2.20[b]	11.01 ± 2.19[b]	9.58 ± 0.82[b]
Diethyl succinate	296.03 ± 47.11[a]	1302.38 ± 267.55[b]	2817.98 ± 305.31[c]	3126.83 ± 141.26[c]
Ethyl 9-decenoate	17.55 ± 3.56[a]	8.59 ± 2.14[a]	8.95 ± 0.95[b]	nd[c]
2-Phenethylacetate	34.48 ± 5.91[a]	28.45 ± 1.95[a]	28.46 ± 4.38[ab]	25.13 ± 4.55[b]
Ethyl phenylacetate	3.15 ± 0.46[a]	3.25 ± 0.70[a]	5.26 ± 0.94[b]	6.31 ± 1.10[b]
Ethyl hydrogen succinate	37.74 ± 2.33[a]	32.74 ± 5.03[a]	46.49 ± 4.63[b]	47.11 ± 2.88[b]
**Tot.**	8195.24 ± 386.94	7290.18 ± 621.76	9103.25 ± 613.40	8814.34 ± 559.53
**Alcohols**				
1-Propanol	24.59 ± 2.07[a]	15.29 ± 1.50[b]	28.84 ± 2.74[c]	nd[d]
Isobutanol	348.47 ± 70.77[a]	349.89 ± 63.72[a]	297.38 ± 53.06[a]	150.72 ± 22.68[b]
1-Butanol	1.01 ± 0.19[a]	14.04 ± 2.23[b]	12.07 ± 1.28[b]	17.53 ± 2.45[c]
Isoamyl alcohol	3161.05 ± 183.76[a]	2840.09 ± 244.77[a]	3110.03 ± 283.28[a]	2827.52 ± 204.91[a]
1-Hexanol	151.29 ± 8.64[a]	132.52 ± 22.39[a]	69.90 ± 6.60[b]	63.97 ± 7.51[b]
1-Octen-3-ol	9.94 ± 0.62[a]	nd[b]	10.30 ± 0.95[a]	8.19 ± 1.22[c]
2-Ethyl-1-hexanol	2.59 ± 0.18[a]	nd[b]	8.17 ± 1.07[c]	nd[b]
1-Octanol	10.09 ± 2.19[a]	nd[b]	nd[b]	nd[a]
Benzyl alcohol	3.29 ± 0.70[a]	7.28 ± 0.86[b]	2.25 ± 0.47[a]	22.42 ± 3.80[c]
Phenylethyl alcohol	3207.95 ± 283.54[a]	2724.46 ± 471.55[ab]	2639.76 ± 469.63[ab]	2179.32 ± 248.23[b]
**Tot.**	6936.94 ± 216.51	6083.57 ± 380.29	6178.67 ± 348.73	5269.66 ± 1053.83
**Acids**				
Acetic acid	166.73 ± 16.11[a]	178.96 ± 26.38[a]	243.80 ± 22.61[b]	334.82 ± 57.13[c]
2-Methyl-hexanoic acid	26.39 ± 2.02[a]	25.11 ± 1.47[a]	30.35 ± 1.32[b]	26.37 ± 2.06[a]
Hexanoic acid	29.04 ± 3.76[a]	52.41 ± 10.76[b]	56.53 ± 9.62[b]	62.33 ± 9.71[b]
Octanoic acid	45.61 ± 1.67[a]	129.76 ± 29.30[b]	163.13 ± 32.01[b]	166.27 ± 25.10[b]
Nonanoic acid	3.47 ± 0.70[a]	3.36 ± 0.48[a]	nd[b]	nd[b]
n-Decanoic acid	26.74 ± 4.85[a]	31.53 ± 3.52[a]	32.73 ± 7.01[a]	62.88 ± 8.48[b]
Benzoic acid	nd[a]	nd[a]	1.59 ± 0.09[b]	6.56 ± 1.41[c]
**Tot.**	297.98 ± 23.60	421.13 ± 35.33	528.14 ± 47.59	659.24 ± 56.71
**Sulphur compounds**				
Dimethyl sulfide	1.34 ± 0.32[a]	3.71 ± 0.74[b]	2.72 ± 0.45[c]	1.55 ± 0.10[a]
Methionol	30.43 ± 4.04[a]	30.33 ± 3.94[a]	16.19 ± 2.15[b]	11.79 ± 0.58[b]
**Tot.**	31.77 ± 3.83	34.04 ± 3.26	33.05 ± 4.28	13.34 ± 0.61
**Aldehydes**				
Furfural	nd[a]	4.17 ± 0.56[b]	5.78 ± 0.46[c]	14.79 ± 0.80[d]
Benzaldehyde	nd[a]	nd[a]	nd[a]	39.91 ± 1.60[b]
5-Hydroxymethylfurfural	nd[a]	nd[a]	nd[a]	2.21 ± 0.20[b]
**Tot.**	nd	4.17 ± 0.56	5.78 ± 0.46	56.90 ± 2.22
**Ketones and lactones**				
Acetoin	5.97 ± 1.25[a]	nd[b]	7.69 ± 1.42[ac]	8.70 ± 1.30[c]
Butyrolactone	7.15 ± 1.30[a]	9.31 ± 1.88[a]	21.30 ± 3.36[b]	24.56 ± 4.39[b]
**Tot.**	13.13 ± 0.09	9.31 ± 1.88	28.99 ± 3.28	33.25 ± 5.29
**Phenols**				
2-Methoxy-4-ethylphenol	nd[a]	2.29 ± 0.37[b]	7.40 ± 1.44[c]	11.38 ± 1.46[d]
4-Ethyl phenol	3.35 ± 0.64[a]	3.35 ± 0.29[a]	24.54 ± 1.18[b]	33.12 ± 6.20[c]
**Tot.**	3.35 ± 0.64	5.64 ± 0.23	31.94 ± 1.71	44.50 ± 7.05
**Terpenes**				
α-Terpinene	7.71 ± 0.78[a]	nd[b]	nd[b]	nd[b]
Terpinen-4-ol	9.24 ± 2.02[a]	nd[b]	nd[b]	nd[b]

α-Terpineol	6.03 ± 1.26^{a}	5.56 ± 0.58^{a}	6.83 ± 0.61^{ab}	nd^{c}
**Tot.**	22.99 ± 2.27	5.56 ± 0.58	6.83 ± 0.61	nd

Results are expressed as RAP = relative peak area (peak area of compound/peak area of internal standard) –> 100 (RAP ± SD). Within each row, the overall means with different superscript letters show a statistically significant difference ($p < 0.05$). The absence of letters indicates non-significant differences between samples. Abbreviations: nd, not detected.
